# A feasibility study using time-driven activity-based costing as a management tool for provider cost estimation: lessons from the national TB control program in Zimbabwe in 2018

**DOI:** 10.1186/s12913-021-06212-x

**Published:** 2021-03-18

**Authors:** J. Chirenda, B. Nhlema Simwaka, C. Sandy, K. Bodnar, S. Corbin, P. Desai, T. Mapako, S. Shamu, C. Timire, E. Antonio, A. Makone, A. Birikorang, T. Mapuranga, M. Ngwenya, T. Masunda, M. Dube, E. Wandwalo, L. Morrison, R. Kaplan

**Affiliations:** 1 College of Health Sciences, Department of Community Medicine, University of Zimbabwe, Harare, Zimbabwe; 2 The Global Fund to Fight TB, HIV and Malaria, Global Health Campus, Chemin du Pommier 40, 1218 Grand-Saconnex, Geneva, Switzerland; 3 Ministry of Health and Child Care, National TB Control Program, Harare, Zimbabwe; 4 Harvard Business School, Boston, MA USA; 5 National Blood Service, Harare, Zimbabwe; 6 Price Waterhouse Coopers (PWC), Harare, Zimbabwe; 7 World Health Organisation, Harare, Zimbabwe

**Keywords:** Tuberculosis, Pathway to care cycles, Provider cost, Time driven activity-based costing

## Abstract

**Background:**

Insufficient cost data and limited capacity constrains the understanding of the actual resources required for effective TB control. This study used process maps and time-driven activity-based costing to document TB service delivery processes. The analysis identified the resources required to sustain TB services in Zimbabwe, as well as several opportunities for more effective and efficient use of available resources.

**Methods:**

A multi-disciplinary team applied time-driven activity-based costing (TDABC) to develop process maps and measure the cost of clinical pathways used for Drug Susceptible TB (DS-TB) at urban polyclinics, rural district and provincial hospitals, and community based targeted screening for TB (Tas4TB). The team performed interviews and observations to collect data on the time taken by health care worker-patient pairs at every stage of the treatment pathway. The personnel’s practical capacity and capacity cost rates were calculated on five cost domains. An MS Excel model calculated diagnostic and treatment costs.

**Findings:**

Twenty-five stages were identified in the TB care pathway across all health facilities except for community targeted screening for TB. Considerable variations were observed among the facilities in how health care professionals performed client registration, taking of vital signs, treatment follow-up, dispensing medicines and processing samples.

The average cost per patient for the entire DS-TB care was USD324 with diagnosis costing USD69 and treatment costing USD255. The average cost for diagnosis and treatment was higher in clinics than in hospitals (USD392 versus USD256). Nurses in clinics were 1.6 time more expensive than in hospitals. The main cost components were personnel (USD130) and laboratory (USD119). Diagnostic cost in Tas4TB was twice that of health facility setting (USD153 vs USD69), with major cost drivers being demand creation (USD89) and sputum specimen transportation (USD5 vs USD3).

**Conclusion:**

TDABC is a feasible and effective costing and management tool in low-resource settings. The TDABC process maps and treatment costs revealed several opportunities for innovative improvements in the NTP under public health programme settings. Re-engineering laboratory testing processes and synchronising TB treatment follow-up with antiretroviral treatments could produce better and more uniform TB treatments at significantly lower cost in Zimbabwe.

## Background

In 2018, Zimbabwe had an estimated Tuberculosis (TB) incidence rate of 210/100,000 population, more than the global average of 130/100,000. Zimbabwe was among the 14 countries with a triple-burden of TB, Tuberculosis/Human immunodeficiency virus (TB/HIV), and multi-drug resistant TB (MDR-TB) [1]. The country had a high TB and HIV co-infection rate of 62% and a treatment coverage of 83% [2]. With nearly one-fifth of undetected TB cases acting as *foci* for community transmission, innovative TB case finding approaches remain were an urgent priority.

Despite high-level political commitment to raise USD15 billion annually for the global TB response, only USD6·9 billion was available from both domestic and international donors in 2018 [3, 4]. In the Zimbabwe context, poor economic performance led to a funding shortfall of more than USD67 million (69%) for meeting the National Tuberculosis Programme (NTP) Strategy Funding requirement for 2017–2020. Despite the funding shortfall, Zimbabwe achieved relatively high treatment coverage of more than 80%. But donors were concerned about the robustness of the costing for NTP’s 2017–2020 strategy. The lack of valid data on TB delivery costs led to incorrect projections and limited optimisation of available resources. This triggered a desire to better measure and understand TB program costs, identify areas that needed improvement in implementation, and guide resource allocation. We applied time driven activity-based costing (TDABC) to calculate the costs of actual healthcare resources used to diagnose and treat TB patients [5–7]. This bottom-up approach, which has had limited application to date in low-income settings, contrasts with the top-down volume-based cost allocation methods used previously in most health settings. This paper illustrates the feasibility of applying TDABC in a low-income setting for costing the care pathway for DS-TB. The study was approved by the Medical Research Council of Zimbabwe (MRCZ/A/2393).

## Methods

### Program set-up

The DS-TB care pathway started with a presumptive TB patient’s initial contact and confirmation as a TB case at the health facility and continued through successful treatment completion. A molecular test using Cepheid GeneXpert was used for the initial diagnosis of all presumptive TB cases. Smear microscopy was used only for treatment monitoring. In 2018, only 125 GeneXpert machines were available for the more than 1000 health facilities in Zimbabwe. Facilities without access to GeneXpert machines relied on private couriers or motorised Environmental Health Technicians (EHTs) to transport sputum sample. A medical officer used chest X-rays for bacteriologically negative but clinically unwell patients to either confirm TB diagnosis or discharge the patient.

Since 2017, Zimbabwe had been using chest X-rays, as a more sensitive initial TB screening tool (Tas4TB) compared to symptoms assessment alone for screening hard-to-reach and high burden populations. Tas4TB was introduced initially in 21 high-burden but low notifying districts. In 2018, Zimbabwe introduced bi-directional screening for diabetes and TB. In this study, facility based TB diagnosis and treatment, was defined as the standard of care.

The treatment regimen for DS-TB consisted of rifampicin (R), isoniazid (H), pyrazinamide (Z) and ethambutol (E) for two (2) months, followed by RH for four (4) months (2RHZE/4RH). Confirmed TB patients were required to visit the health facility once every 2 weeks to collect medicines and assess treatment efficacy. At 2–3 months, 5 and 6 months, repeat sputum specimens were collected as part of treatment monitoring and to confirm cure.

### Study sites

We selected nine (9) health facilities (study sites), considering disease burden and level of care managed by either the Ministry of Health and Child Care (MoHCC) or the Ministry of Local Government. Two provincial hospitals **(**Chinhoyi and Gwanda), two district hospitals (Maphisa and Banket), four urban polyclinics (Rutsanana, Dzivarasekwa, Mzilikazi and Princess Margaret) and one Tas4TB mobile clinic were selected for the study.

### Costing approach and implementation

A multidisciplinary team from NTP, University of Zimbabwe, College of Health Sciences, (UZCHS), Price Waterhouse Coopers Advisory Services (PWC), Harvard Business School (HBS) and the Global Fund (GF) conducted the study using TDABC. Capacity building was achieved through training of national partners on TBABC, joint development of process maps and tools, and pilot testing facilitated by GF and HBS.

Process maps for TB care pathways were developed iteratively using a combination of diagnostic algorithms, observations of workflows at the facilities and discussions with health care workers. Diagnostic maps captured the care pathway processes from the time a patient presented with signs and symptoms to confirmation of TB. Treatment maps captured the patient care pathway processes from notification of a confirmed TB case to completion of recommended treatment, which is, 6 months for DS-TB. Tas4TB maps described the process from site identification, demand creation, TB symptom screen using a WHO symptom screening tool, digital chest X-ray, Xpert MTB/Rif tests for presumptive cases, and treatment initiation in communities.

We observed health care workers providing TB care services at different TB care pathway stages, and documented the personnel type performing the task and the time taken per stage as described by Kaplan and Porter [7].

The USD/minute capacity cost rate (CCR) was measured for each resource type at a clinic (personnel or equipment) by dividing the annual cost of the resource by its annual practical capacity. Practical capacity calculation for personnel was based on five working days per week for each health care worker adjusted to exclude days used for vacation, continuing professional development, sick leave and health breaks. The team estimated number of minutes available each day of each personnel type, and multiplied this quantity by number of working days per year to obtain the total minutes available per year per person. Salary data for each cadre of health care worker was obtained from the HR Departments in the MoHCC and Local Government Authorities.

A similar method was used to calculate the CCRs for equipment and space. We assumed equipment utilization of 85% with 15% down time for repairs, maintenance, and scheduled calibrations [8]. Cost of equipment was based on most recent procurement data supported by government or donors and useful life of equipment. Useful life of equipment was estimated as 10 years for X-ray machines and vans; 5 years for GeneXpert, full blood count, chemistry and audiometer, and 7 years for the digital X-ray machines.

We measured square metres within a health facility dedicated to delivering TB care and time taken to provide TB services. In case of shared space, estimates were used. Cost of space was based on replacement cost per square metre, useful life (depreciation), plus annual operating expenses as defined in the MoHCC guidelines and costing of the National Health Strategy (NHS), 2015–2020.

Time taken by personnel or equipment at each step of the process map was measured for individual patients and averaged to calculate the average time per patient for each activity step. This time was then multiplied by the personnel or equipment’s capacity cost rate (CCR) to calculate the resource’s cost to perform each treatment step.

In addition to the cost of human resources (HR) and equipment, the analysis calculated the costs of utilities (water and electricity supply), medicines, including anti-TB medicines, laboratory supplies, and other consumables using data from the facilities and the NTP at central level. The overhead cost related to program coordination by the NTP Unit within MoHCC, was assumed to be independent of the quantity and mix of patients treated and was excluded from the analysis.

The final calculated provider unit costs per patient, measured in United States Dollars (USD), were aggregated by cost domain and facility. Using the cost of diagnosis and treatment of one TB patient per facility, we estimated the total cost per facility using routinely reported facility data for 2017. This assessed cost variation by facility.

### Data collection and management

The study was conducted from August 2018 to January 2019. The project team trained registered nurses, at each TB facility, to be the research assistants (RA) to collect data. The RAs were assigned to facilities away from their usual workstations to minimise bias. Training and supervision of data collection were provided by the central team of experts from the NTP, UZCHS, PwC, HBS and GF.

Data collection tools were pre-tested in four non-participating facilities, two rural and two urban. One of the rural facilities was a district hospital. The tools were adjusted accordingly after the pilot and before field work. The tools were programmed and uploaded into REDCap (Research Electronic Data Capture; https://projectredcap.org/software/), a data collection, transmittal and storage open source software. Data were transmitted electronically to the central level where the study biostatistician provided data quality checks and appropriate feedback to the RAs.

### Study participants

Health care workers (HCWs) and facility managers from two provincial hospitals, two district hospitals and four clinics, were interviewed as key informants. All the four clinics were from the two metropolitan cities of Harare, the capital city and Bulawayo, the second largest city in Zimbabwe. These were high volume facilities that ensured adequate tuberculosis patients to interview until saturation. Each HCW-patient pair was observed during diagnosis, care, and treatment to collect information on time and resources used step by step. We observed 3 to 7 HCW-patient pairs to reach saturation and get an optimal average time for each stage. Informed consent was obtained from all research participants including patients.

## Results

Six hundred and seventy (670) observations for health care worker-patient pairs were conducted across nine sites. We interviewed 116 key informants.

### Availability of TB services by facility

All four hospitals had capacity to offer chest X-ray, Xpert MTB/Rif/Ultra, full blood count, and chemistry services. One urban clinic, Dzivarasekwa, had a GeneXpert machine. The three clinics without GeneXpert machines transported sputum specimen to nearest GeneXpert sites. All facilities had microscopy services for treatment monitoring. Facilities with no X-ray, chemistry and full blood count services referred patients to hospitals offering the services. Hospitals provided entire cascade of care from diagnosis, treatment follow-up, to treatment outcomes evaluation. Integrated TB/HIV services were available in all sites (Table 1). The Tas4TB used a mobile van equipped with chest X-ray machines for TB screening. Clients who were positive on chest x-ray and symptom screen, had sputum collected and referred to nearest facility with GeneXpert machine.

**Table 1:** Equipment availability (TB Services) by facility, Zimbabwe TDABC, 2018

	Banket	Chinhoyi	Dzivarasekwa	Gwanda	Maphisa	Mzilikazi	Princess Margaret	Rutsa-nana	Tas4TB
Digital CXR	1	2	0	1	1	0	0	0	3
GeneXpert	1 GX4	1 GX4	1 GX4	1 GX4	1 GX4	0	0	0	3 GX4
FBC Machine	2	3	0	2	2	0	0	0	0
Chemistry Analyser	1	3	0	2	1	0	0	0	0
Microscope	1	2	1	2	2	3	0	1	4
TSH Machine	0	1	0	1	0	0	0	0	0
Audiometry Machine	0	1	0	0	0	0	0	0	0
TB Mobile Van	0	0	0	0	0	0	0	0	1

### Diagnosis, treatment and care pathways for DSTB

**Fig. 1:**
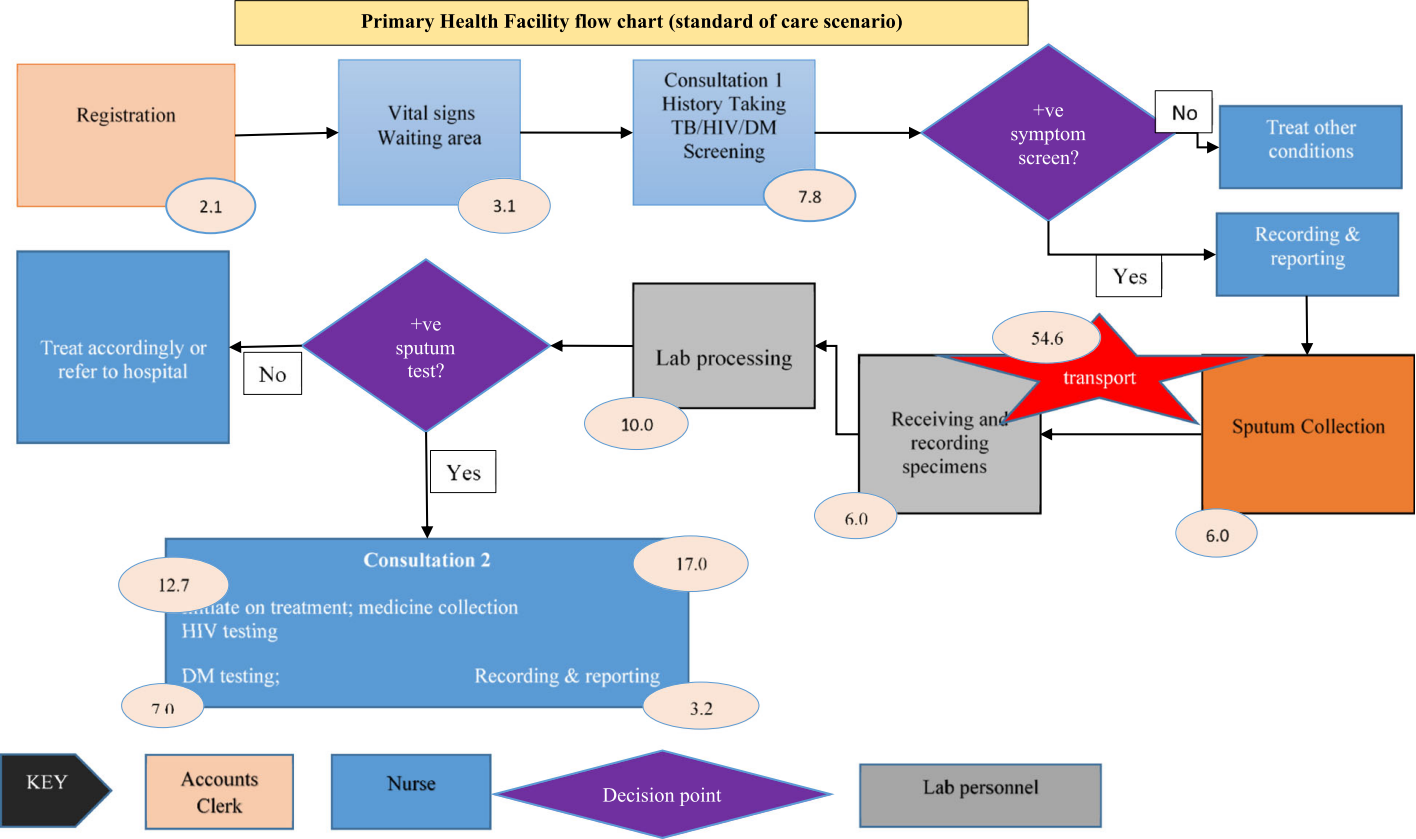
Primary care facility (clinic) TB Standard of Care pathway, 2018*.* HIV=Human immune-deficiency virus; DM = Diabetes mellitus; EHT = Environmental health technician; TB = Tuberculosis; Vitals (BP=Blood Pressure; Ht = Height; Wt = Weight; Temp = Temperature); Lab = Laboratory; +ve = Positive

**Fig. 2:**
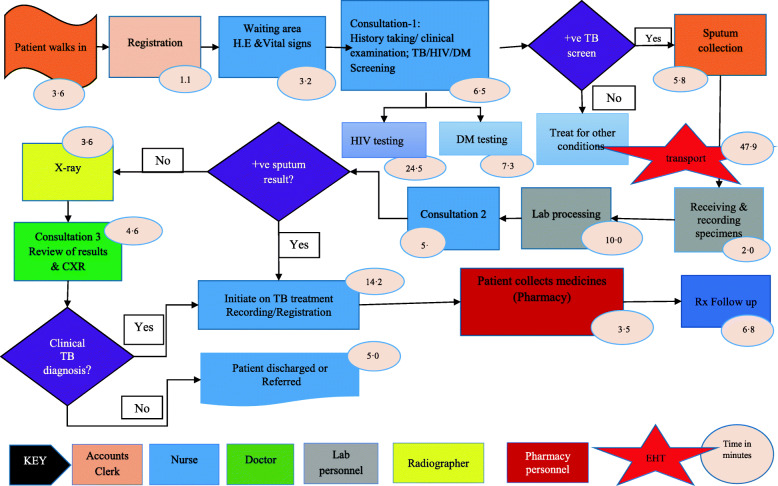
Hospital TB standard of care pathway, Zimbabwe TDABC, 2018. HIV=Human immune-deficiency virus; DM = Diabetes mellitus; EHT = Environmental health technician; TB = Tuberculosis; Vitals (BP=Blood Pressure; Ht = Height; Wt = Weight; Temp = Temperature); Lab = Laboratory; +ve = Positive

**Fig. 3:**
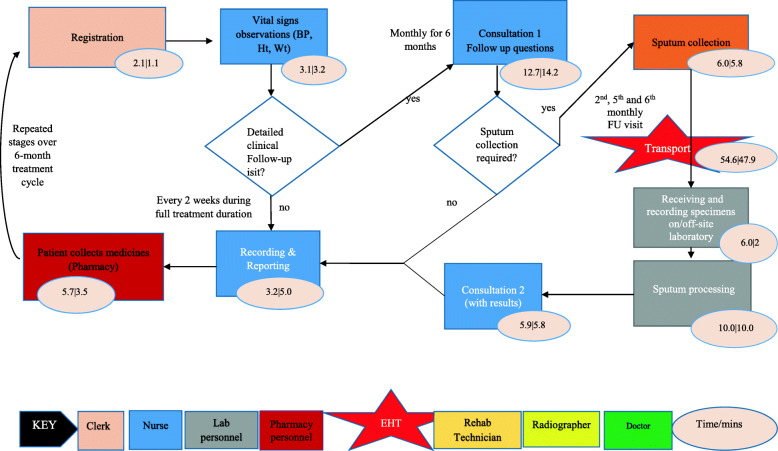
TB Treatment follow-up pathway, (Clinic and Hospital) 2018. EHT = Environmental health technician; TB = Tuberculosis; BP=Blood Pressure; Ht = Height; Wt = Weight; Temp = Temperature; Lab = Laboratory; DSTB = drug sensitive TB; DRTB = drug resistant TB; U&E + Creat = urea and electrolytes + creatinine; ECG = electrocardiogram; FU = follow up; qns = questions; LFT = liver function test; TSH = thyroid stimulating hormone

**Fig. 4:**
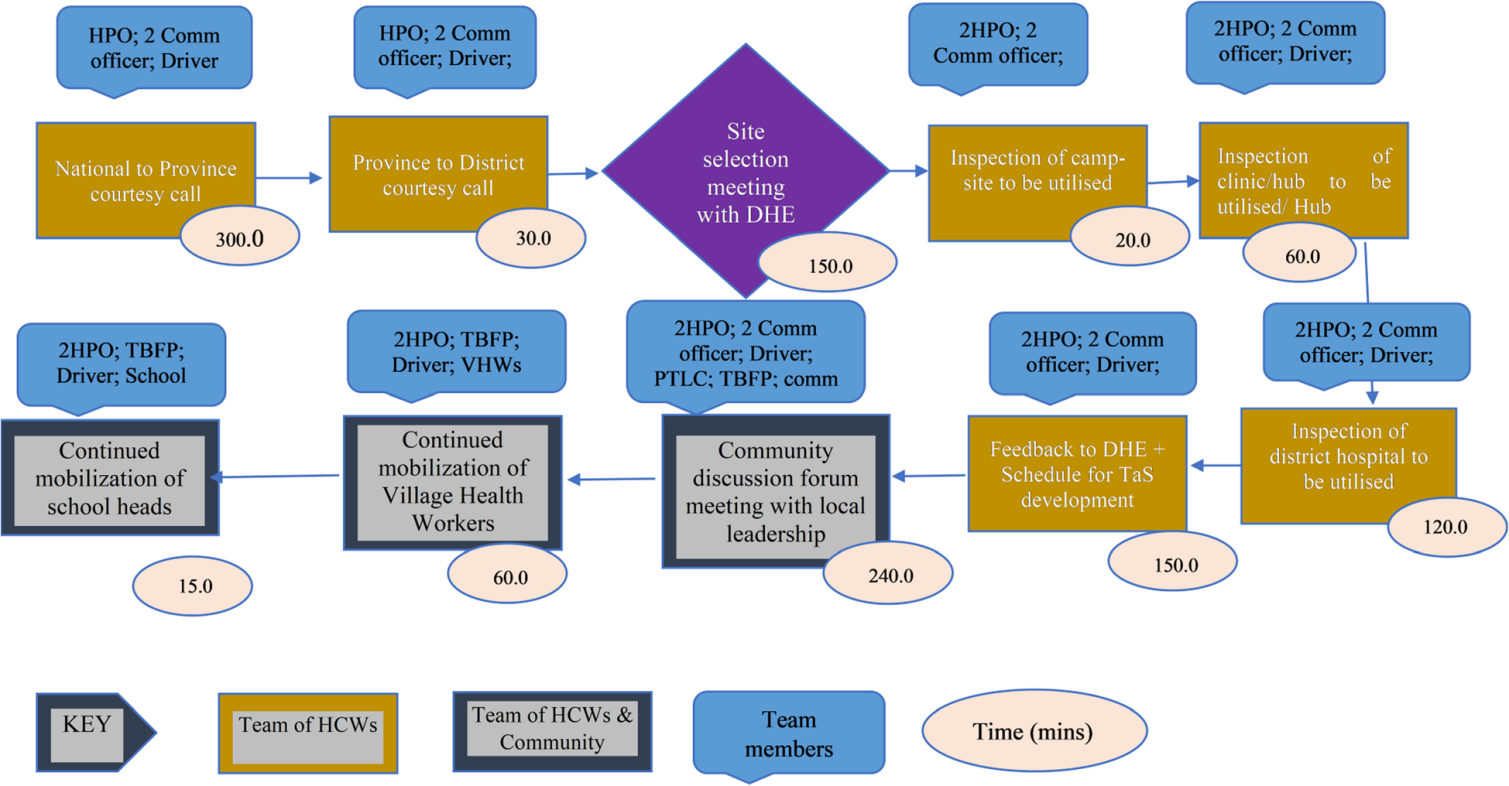
Tas4TB Demand creation TB Case finding pathway, Zimbabwe TDABC, 2018

**Fig. 5:**
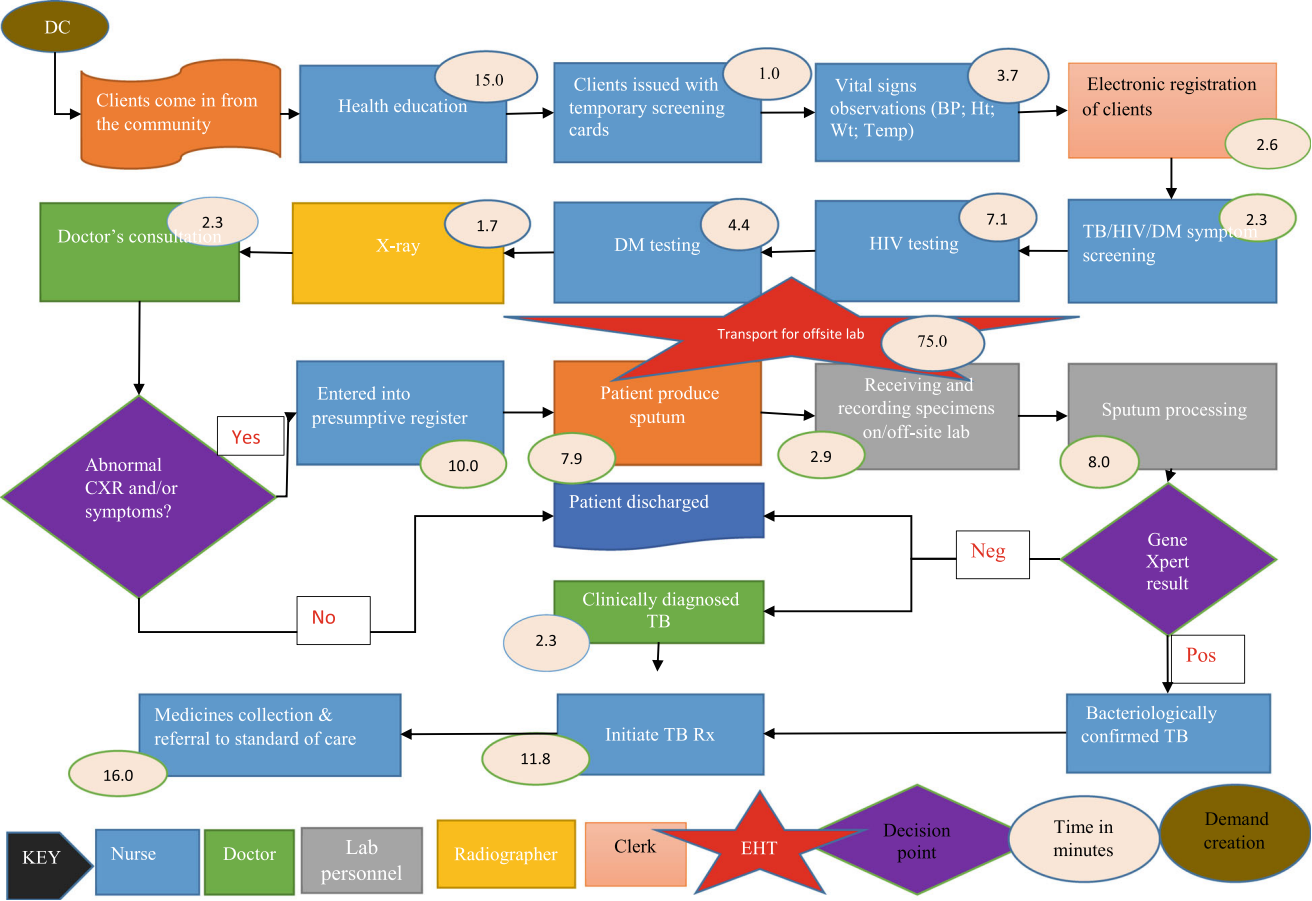
Tas4TB Active TB Case finding pathway, Zimbabwe TDABC, 2018. DC=Demand creation; HIV=Human immune-deficiency virus; DM = Diabetes mellitus; EHT = Environmental health technician; CXR = Chest X-ray; TB = Tuberculosis; BP=Blood Pressure; Ht = Height; Wt = Weight; Temp = Temperature; Lab = Laboratory; Pos = Positive; Neg = Negative; Rx = Treatment

Twenty-five stages of the care pathway were identified from the process maps. The stages were relatively similar across facilities except for Tas4TB (Figs. 1, 2, 3, 4 and 5). Community Tas4TB included demand creation stage and excluded treatment follow-up. All TB confirmed patients diagnosed through Tas4TB were referred to the nearest facility for treatment follow up.

The different facilities had considerable variations in the personnel types that performed registration, vital signs, treatment follow-up, medicines dispensing and sample processing (Tables 2 and 3). Table 2 shows that a clerk performed the patient registration service. In all facilities, a nurse provided initial and second consultations. However, third consultation, was almost always done by the medical officer. Table 3 shows that nurses provided most of the services, except for laboratory testing performed by a laboratory scientist or technologist. Five facilities used nurse aides, and four facilities used nurses for vital signs. Diabetes Mellitus (DM) and HIV testing were embedded in the diagnosis stage care cycle. Sub-contracted private courier performed sample transportation in Harare City; EHTs did this task at all other facilities. After treatment initiation, the nurse reviewed DSTB patients and monitored adverse drug events every 2 weeks for 6 months during appointments scheduled for patients'drug pick up. The last treatment monitoring follow-up review was done by medical officers to assess treatment outcome status.

**Table 2:**
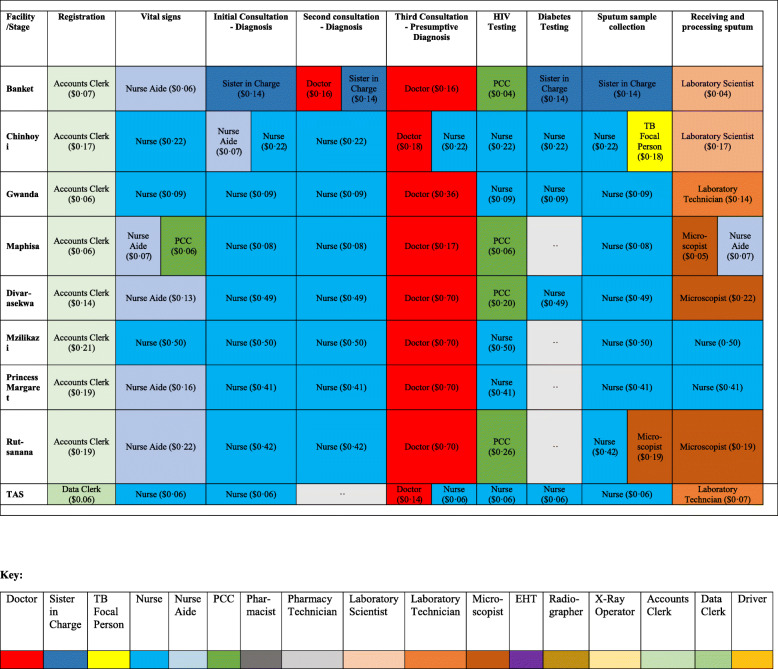
Personnel capacity cost rates by facility by stage, Zimbabwe TDABC, 2018

**Table 3:**
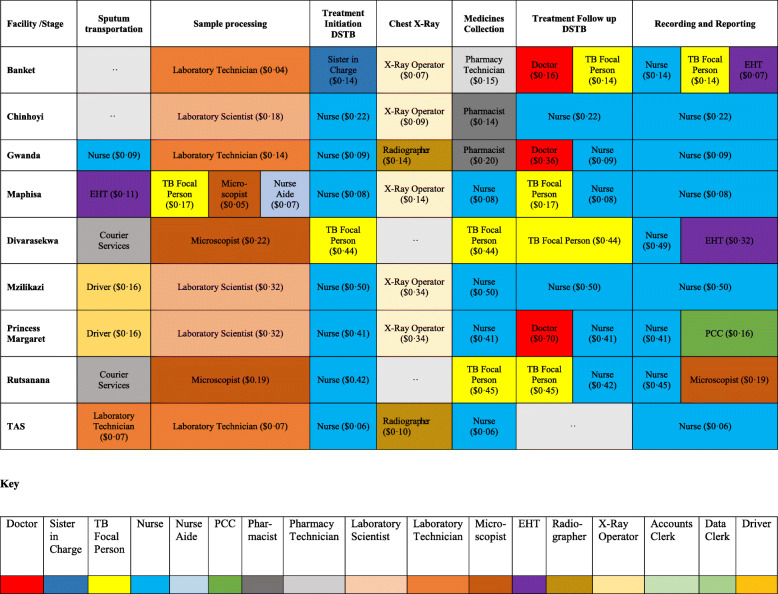
Personnel capacity cost rates by facility by stage, Zimbabwe TDABC, 2018

### Average time spent on each stage by facility type

Sample processing and transportation and HIV testing required the longest average times for health care workers, range, 64–110 (Table 4). Hospitals took more time processing samples and testing for HIV compared to clinics. The services with the least times were registration and vital signs, range 0.9–2.6 and 3–3.7 respectively. Tas4TB, used in remote locations far from GeneXpert sites, had longer sample transportation (75 min) than district hospitals (69 min) and clinics (55 min). The recording and reporting of data, as well as treatment initiation took much longer in Tas4TB than in standard of care (10 vs 4 and 16 vs 4 respectively). First consultation during diagnosis phase was longer in clinics compared to both Tas4TB and hospitals (8 vs 2 vs 6) respectively. Tas4TB, a new concept in Zimbabwe, used a recording and reporting tool with 66 variables that took longer to complete than the reporting at the other three settings. There were no variations in time taken by presence of co-morbidities or gender across all the stages and DM testing. The average time for HIV testing was high in district hospitals compared to both urban clinics and hospitals. Efficiencies were realized for patients with known comorbid conditions (such as HIV) since they did not need separate counselling and testing sessions for those conditions.

**Table 4:** Average observation time in minutes for each stage by facility type, co-morbidity and gender

Stage	Facility type				Co-morbidity		Gender	
	Provincial Hospital	District Hospital	Urban Polyclinic	Tas4TB Rural	Yes	No	Female	Male
Registration	1.5 (12)	0.9 (21)	2.1 (27)	2.6 (7)	3 (4)	2.8 (12)	1.7 (41)	1.7 (26)
Vital signs	3.1 (10)	3.3 (17)	3.1 (27)	3.7 (7)	4.5 (2)	3.5 (22)	2.9 (34)	3.6 (27)
Initial Consultation	7.1 (8)	6.2 (17)	7.8 (22)	2.3 (7)	6.1 (19)	6.6 (32)	6.7 (29)	6.3 (25)
HIV Testing Services (HTS)	19.7 (7)	26.6 (16)	17 (26)	7.1 (7)	13.8 (5)	20.1 (39)	17.9 (37)	20.7 (19)
Diabetes Mellitus Testing Services (DM)	11.7 (3)	3 (3)	7 (2)	4.4 (7)	6.7 (6)	5.4 (9)	6 (10)	5.8 (5)
Recording and reporting	3.6 (5)	5.3 (19)	3.2 (31)	10 (5)	4.4 (23)	4.7 (35)	3.9 (31)	5.1 (28)
Sputum Sample Collection for TB diagnosis	5.5 (11)	5.9 (13)	6 (24)	7.9 (7)	5.8 (25)	6.4 (25)	5.8 (33)	6.6 (23)
Sputum sample transportation	3.2 (6)	68.5 (13)	54.6 (16)	75 (7)	51.3 (10)	47.7 (11)	58.4 (17)	62.1 (21)
Receiving and Initial registration Sputum Specimens	2.1 (11)	1.7 (3)	6 (20)	2.9 (7)	3.4 (16)	2.8 (14)	3 (21)	5.3 (19)
Sample processing and registration of results	88.2 (10)	110.1 (13)	63.5 (22)	84.8 (8)	42.9 (17)	59.8 (13)	66.7 (21)	87.7 (29)
Second Consultation, with sputum results	5 (9)	6.3 (16)	5.9 (21)	..	6.1 (18)	5.9 (24)	5.7 (28)	6.1 (18)
Chest X-ray	3.2 (6)	3.9 (11)	7.3 (4)	1.7 (7)	2.6 (8)	3.1 (14)	3.9 (18)	3.3 (10)
Consultation after Chest X-ray	..	..	..	2.3 (14)	1.8 (6)	2.6 (7)	2.3 (13)	2 (1)
Third Consultation, for sputum negative presumptive cases	5.3 (9)	3.9 (11)	11 (3)	..	6.6 (9)	4.3 (13)	5.1 (12)	5.7 (11)
Treatment initiation DS-TB)	15.9 (7)	13.4 (14)	12.7 (6)	11.8 (5)	15.5 (17)	11.4 (15)	15.7 (12)	12.3 (20)
Medicines Collection (anti-TB and ancillary)	2.5 (10)	4.1 (19)	5.7 (28)	16 (3)	6.2 (29)	4.4 (29)	6.4 (22)	4.4 (38)
Treatment follow-up	7.4 (10)	6.4 (14)	6.6 (27)	..	6.8 (27)	6.5 (24)	7.2 (22)	6.4 (27)

### Space and equipment capacity cost rate, Zimbabwe TDABC, 2018

Table 5, shows the different cost capacity rates of the space used to provide TB services by facility and type of activities being performed. The consulting rooms for hospitals had higher space CCR compared to clinics. Capacity cost rates for waiting area, laboratory, and pharmacy were the highest across all facilities. Chinhoyi Hospital had the highest CCR for waiting area (USD0·98), pharmacy (USD0·105) and laboratory (USD0·145). The waiting area CCR for Dzivarasekwa clinic was similar to that of Maphisa and Banket district hospitals, USD0·05. In other clinics, some TB care activities were being provided in the same space, for example, waiting area and first consulting rooms. Tas4TB had the lowest space CCR of all facilities. Capacity cost rate for GeneXpert was USD0·05 and USD0·19 for chest X-ray· The most expensive equipment per patient was the TB mobile van, with a CCR of USD0·57, given the added fuel and vehicle costs.

**Table 5:** Space and Equipment capacity cost rate, Zimbabwe TDABC, 2018

Facility	Type of Space	Activity happening in the identified Space	Actual Area Used (sqm)	CCR/ minute (USD)
Banket District Hospital	Waiting room	Registration, Vital Signs, Initial Consultation, Recording and reporting	364.5	**0.048**
	Consulting Room Nursing	Third Consultation sputum negative presumptive cases, Treatment Initiation for DSTB, Initial Consultation, Second Consultation with sputum results, Sputum sample collection for TB diagnosis, Medicines Collection, HIV Testing Services, Recording and Reporting, Treatment Follow up DSTB, Diabetes Mellitus Testing Services	27	**0.004**
	Consulting Room - Doctor	Second Consultation with Sputum results, Third Consultation for sputum negative presumptive cases, Treatment follow up DSTB	54	**0.007**
	Laboratory	Sample processing and registration of results, Chest X-Ray	90	**0.012**
	Pharmacy	Medicines Collection (Anti-TB and Ancillary)	90	**0.012**
Chinhoyi Provincial Hospital	Waiting room	Registration, Treatment Follow up DSTB	750	**0.098**
	Consultation Room	Second Consultation with Sputum results, treatment follow up DSTB	60	**0.008**
	Consulting Room Nursing	Initial Consultation, Second Consultation with Sputum results, Treatment Initiation DSTB, Treatment Follow up DSTB, Sputum Sample collection for TB diagnosis, Vital signs, HIV Testing Services, Diabetes Mellitus Testing Services,	60	**0.008**
	Consulting Room - Doctor	Third consultation for sputum negative presumptive cases	60	**0.008**
	Laboratory	Receiving and initial registration of sputum specimens, Treatment Follow up, Sample processing and registration of results	1108	**0.145**
	Pharmacy	Medicines Collection (Anti-TB and Ancillary)	800	**0.105**
Gwanda Provincial Hospital	Waiting room	Registration, Vital Signs	42.5	**0.006**
	Consultation Room	Initial Consultation		**0.004**
	Consulting Room Nursing	Initial Consultation, Second Consultation with Sputum results, Treatment Initiation DSTB, Treatment Follow up DSTB, Sputum Sample collection for TB diagnosis, HIV Testing Services, Recording and Reporting	31.5	**0.004**
	Consulting Room - Doctor	Third consultation for sputum negative presumptive cases, Treatment follow up DSTB	10	**0.001**
	Laboratory	Receiving and initial registration of sputum specimens, Sample processing and registration of results, sputum sample transportation	12.6	**0.002**
	Pharmacy	Medicines Collection (Anti-TB and Ancillary)	10.25	**0.001**
	X-ray Room	Chest X-ray		
Maphisa District Hospital	Waiting room	Registration, Vital Signs, Initial Consultation, HIV Testing Services, Treatment follow up	364.5	**0.048**
	Consultation Room - Nurse	Initial Consultation, Second Consultation with Sputum Results, Sputum sample collection for TB Diagnosis, HIV Testing Services, Treatment follow up DS-TB, Recording and Reporting	27	**0.004**
	Consulting Room - Doctor	Initial Consultation, Treatment Initiation DS-TB, Recording and Reporting	54	**0.007**
	Laboratory	Receiving and initial registration of sputum specimens, Sample processing and registration of results, Sputum sample transportation, Recording and Reporting	90	**0.012**
	Pharmacy	Medicines Collection (Anti-TB and Ancillary)	90	**0.012**
Dzivarasekwa Clinic	Waiting room	Registration, Vital Signs, and Treatment follow up	342	**0.045**
	Consultation Room	HIV Testing Services, Diabetes Mellitus Testing Services, Treatment Follow up DS-TB, Recording and Reporting		**0.002**
	Consulting Room Nursing	Initial Consultation, Second Consultation with sputum results, Diabetes Mellitus Testing Services, Recording and Reporting	18	**0.002**
	Consulting Room - Doctor	Third Consultation for sputum negative presumptive cases	18	**0.002**
	Laboratory	Receiving and initial registration of sputum specimens, sample processing and registration of results, Sputum sample collection for TB diagnosis, Recording and Reporting	16	**0.002**
	Pharmacy	Medicines Collection (Anti-TB and Ancillary), Treatment follow up DS-TB	6	**0.001**
Mzilikazi Clinic	Waiting room	Registration, Vital Signs, HIV Testing Services, Treatment Follow up	16.8	**0.004**
	Consultation Room	HIV Testing Services, Treatment Follow up DS-TB, Second Consultation with sputum results, Recording and Reporting	16.8	**0.004**
	Consulting Room Nursing	Initial consultation, Sputum sample collection for TB diagnosis, Second Consultation with sputum results, Recording and Reporting, Medicines collection (Anti-TB and Ancillary)	29.2	**0.004**
	Consulting Room - Doctor	Third Consultation for sputum negative presumptive cases	15.2	**0.002**
	Laboratory	Thorngroove Laboratory	69	**0.009**
	Pharmacy	Medicines Collection (Anti-TB and Ancillary), Treatment follow up DS-TB	12	**0.002**
	X-ray Room	Khami Clinic		
Princess Margaret Clinic	Waiting room	Registration, Vital Signs	41.2	**0.005**
	Consulting Room Nursing	Initial consultation, second consultation with sputum results, HIV testing services, Sputum sample collection for TB diagnosis, Treatment Initiation DS-TB, Treatment follow up DS-TB, Recording and Reporting, Medicines collection (Anti-TB and Ancillary)	104.2	**0.014**
	Consulting Room - Doctor	Third Consultation for sputum negative presumptive cases, Treatment follow up DS-TB	13.25	**0.002**
	Laboratory	Thorngroove Clinic, Recording and Reporting	69	**0.009**
	Pharmacy		12	**0.002**
	X-ray Room	Khami Clinic		
Rutsanana Clinic	Waiting room	Registration, Vital Signs	106	**0.014**
	Consultation Room	HIV Testing Services, Treatment follow up DS-TB		**0.002**
	Consulting Room Nursing	Initial consultation, second consultation with sputum results, Treatment initiation DS-TB, Sputum sample collection for TB diagnosis, Treatment follow up DS-TB, Recording and reporting	12	**0.002**
	Consulting Room - Doctor	Sputum sample collection for TB diagnosis	12	**0.002**
	Laboratory	Receiving and initial registration of sputum specimens, sample processing and registration of results, Medicines collection (Anti-TB and Ancillary), Recording and Reporting	14	**0.002**
	Pharmacy	Medicines collection (Anti-TB and Ancillary)	12	**0.002**
Tas4TB	Waiting room	Registration, Vital Signs, Sputum sample collection for TB diagnosis, Treatment Initiation DS-TB, Medicines Collection (Anti-TB and Ancillary), Recording and Reporting	60	**0.008**
	Consultation Room	Initial Consultation, Diabetes Mellitus Testing Services, HIV Testing Services		**0.004**
	Consulting Room Nursing	Initial consultation, HIV Testing Services, Diabetes Mellitus Testing Services, Recording and Reporting, Treatment Initiation DS-TB, Doctor’s consultation after CXR	30	**0.001**
	Consulting Room - Doctor	Initial Consultation, Chest X-Ray, Treatment Initiation DSTB, Doctor’s consultation after CXR	5	**0**
	Laboratory	Receiving and initial registration of sputum specimens, sample processing and registration of results,	9.36	**0**
Digital CXR				**0·19**
GeneXpert				**0·05**
FBC Machine				**0·06**
Chemistry Analyser				**0·09**
Microscope				**0·01**
TSH Machine				**0·01**
Audiometry Machine				**0·01**
TB Mobile Van				**0·57**

### The cost of care for DS-TB

The average cost per patient of the entire care pathway was USD324 for all facilities (Table 6). The average cost was higher in clinics (USD392) compared to hospitals (USD256). The cost for hospitals ranged from USD239 to USD272 while clinics ranged from USD335 to USD489. Mzilikazi and Princess Margaret clinics had costs of over USD400, due to high unit cost for sample transportation and HIV testing.

**Table 6:** Cost of DS-TB Patient diagnosis and treatment in Zimbabwe, TDABC, 2018

Key Stage	Step No.	Step Description	Facility / Stage											
			Banket District Hospital	Chinhoyi Provincial Hospital	Gwanda Provincial Hospital	Maphisa District Hospital	Unit Cost Hospitals	Dzivar-asekwa Clinic	Mzilikazi Clinic	Princess Margeret Clinic	Rutsanana Clinic	Average cost (clinics)	Average cost (all health facilities)	Unit Cost Tas4TB
Diagnostic	Step 1(a)	Demand Creation *(only applicable for Mobile/Targeted Active Screening - TAS4TB)*	–	–	–	–	–	–	–	–	–	–	**–**	89.21
	Step 1(b)	Registration (Diagnostic)	0.12	0.27	0.15	0.11	**0.16**	0.72	0.24	0.29	0.39	**0.41**	**0.29**	0.17
	Step 2	Vital signs	0.38	1.15	0.25	0.35	**0.53**	0.54	0.69	0.53	0.60	**0.59**	**0.56**	0.27
	Step 3(a)	First Consultation	0.99	2.69	0.49	0.85	**1.26**	3.38	6.37	2.75	2.50	**3.75**	**2.50**	0.16
	Step 3(b)	Co-infection/morbidity testing: HIV Testing Services (HTS)	2.70	5.51	2.75	1.97	**3.23**	3.88	11.84	11.33	2.70	**7.44**	**5.33**	1.42
	Step 3 (c)	Co-infection/morbidity testing: Diabetes Mellitus Testing Services (DM)	0.42	2.67	1.12	0.26	**1.12**	3.47	–	–	–	**0.87**	**0.99**	0.3
	Step 4(a)	Laboratory: Sputum Collection	1.94	2.70	1.26	1.08	**1.75**	4.05	3.62	2.56	4.52	**3.69**	**2.72**	2.47
	Step 4(b)	EHT: Sputum sample transportation *(only applicable for sites without diagnostic equipment)*	0.44	–	0.28	0.30	**0.26**	–	14.93	9.87	–	**6.20**	**3.23**	4.92
	Step 5	Laboratory: Receiving and initial registration of Sputum Specimens	0.44	0.47	0.22	0.20	**0.33**	0.87	5.01	4.09	0.74	**2.68**	**1.51**	0.19
	Step 6	Laboratory: Sputum Processing & Recording Results	17.13	16.55	20.10	16.74	**17.63**	15.41	24.06	24.06	13.96	**19.37**	**18.50**	19.3
	Step 7	Second Consultation (+ve/−ve sputum results)	2.00	1.55	0.25	0.64	**1.11**	4.37	2.10	1.23	2.75	**2.61**	**1.86**	..
	Step 8	Chest X-ray (−ve sputum results)	34.81	34.36	34.36	34.22	**34.44**	–	37.07	37.07	–	**18.54**	**26.49**	33.5
	Step 9	Third Consultation (CXR results)	0.71	3.67	1.26	0.70	**1.59**	9.13	6.32	7.72	7.01	**7.54**	**4.56**	0.47
Treatment Initiation	Step 10 (a)	Recording/Reporting (Treatment Initiation - DS TB)	1.62	1.31	0.35	0.50	**0.94**	3.12	1.60	2.06	1.64	**2.11**	**1.52**	0.76
	Step 10 (b)	Consultation (Treatment Initiation - DS TB)	1.51	2.13	1.93	0.18	**1.44**	5.30	10.11	8.25	3.48	**6.78**	**4.11**	..
	Step 11	Medicines Collection	0.55	0.83	0.44	0.17	**0.50**	1.90	2.03	4.49	1.83	**2.56**	**1.53**	..
Treatment Follow-Up: No. of visits = 12	Repeat: Step 10	Registration (Treatment follow up - DS TB)	0.92	2.08	1.69	0.77	**1.37**	6.60	2.86	3.33	4.34	**4.28**	**2.82**	..
	Repeat: Step 2	Vital signs (Treatment follow up - DS-TB)	2.71	9.53	2.77	3.09	**4.53**	4.72	8.09	6.10	6.78	**6.42**	**5.47**	..
	Repeat: Step 7	Consultation (Treatment follow up - DS-TB)	1.28	21.38	34.65	15.03	**18.08**	36.90	32.29	97.70	77.16	**61.01**	**39.55**	..
	Repeat: Step 11	Medicines Collection	39.59	39.04	38.62	35.22	**38.12**	56.12	57.60	85.48	55.29	**63.62**	**50.87**	..
		Recording and Reporting	13.64	10.59	3.84	3.73	**7.95**	35.39	18.91	23.94	19.17	**24.35**	**16.15**	..
Treatment Follow Up: No. of sputum test = 2	Repeat: Step 4(a)	Laboratory: Sputum Collection	3.86	5.32	2.48	2.13	**3.45**	8.08	7.19	5.01	9.03	**7.33**	**5.39**	..
	Repeat: Step 4(b)	EHT: Sputum sample transportation *(only applicable for sites without diagnostic equipment)*	–	–	0.56	0.61	**0.29**	–	29.86	19.74	–	**12.40**	**6.35**	..
	Repeat: Step 5	Laboratory: Receiving and initial registration of Sputum Specimens	0.87	0.95	0.43	0.40	**0.66**	1.75	10.03	8.19	1.49	**5.36**	**3.01**	..
	Repeat: Step 6	Laboratory: Sputum Processing & Recording Results	80.50	82.74	82.25	85.09	**82.64**	83.72	85.81	85.81	83.21	**84.63**	**83.64**	..
	Step (unspe-cified)	Overheads	37.38	24.93	32.56	34.17	**32.26**	37.12	37.90	37.37	35.97	**37.09**	**34.68**	..
**SUMMARY PATIENT COST**														
Cost of diagnostic			62.07	71.58	62.49	57.45	**63.40**	45.83	112.24	101.50	35.19	**73.69**	**68.54**	153.14
Cost of treatment initiation			3.67	4.27	2.72	0.84	**2.88**	10.32	13.73	14.80	6.94	**11.45**	**7.16**	..
Cost of treatment follow up			180.75	196.56	199.84	180.24	**189.35**	270.39	290.55	372.66	292.43	**306.51**	**247.93**	..
Cost of treatment (initiation & follow up)			184.42	200.83	202.56	181.08	**192.22**	280.71	304.28	387.46	299.37	**317.96**	**255.09**	..
Total Patient Cost per facility			246.49	272.42	265.05	238.53	**255.62**	326.53	416.53	488.97	334.56	**391.65**	**323.63**	..

The diagnostic cost was USD69 and treatment cost was USD255 per patient. Treatment cost ranged from USD181 to USD387 per patient. The average diagnostic and treatment costs per patient were 1·2 and 1·7 times higher in clinics than in hospitals. The average cost of HIV testing per patient was higher in clinics (USD7) compared to hospitals (USD3), due to the type of HCW. The diagnostic costs in clinics were high due to the transportation of sputum samples from clinics to district hospitals as well as use of qualified nurses at clinics to perform HIV testing. Sample processing cost per patient during treatment stage was higher compared to diagnosis stage (USD84 versus USD19). These high costs were driven by repeat tests during treatment follow-up visits. Treatment costs were increased by the repeat two weekly visits to collect anti-TB medication.

Table 7 describes the contribution of each domain to the total cost of TB diagnosis and treatment. On average and across all facilities, the major cost drivers were HR (40%) and laboratory (37%). Cost of diagnosis and treatment of TB was higher in urban clinics compared to hospitals due to variation in cost of labour. The HR costs were lower in hospitals USD56 vs USD204 compared to clinics. Laboratory costs were higher in hospitals USD127 vs USD111 in clinics since most of the laboratory work was hospital based. Nurses working in urban polyclinics were paid more, contributing to the higher costs of providing TB services at clinics compared to hospitals (average USD0·46 vs USD0·08 per minute). Medical officers from urban clinics were expensive compared to district and provincial hospitals average (USD0·50 vs USD0·16). The overall cost of treatment was pushed up by the number of follow-up visits to facilities (of up to six) made by patients. Half of the visits are only to collect the next 2 weeks of refills, with no clinical assessment beside vitals.

**Table 7:** Average Unit cost (USD) by type of facility and domain

	Human Resources	Medicines	Equipment	Laboratory Consumables	Space	Overhead	Average cost per patient
Hospitals	56.00	33.53	4.09	127.45	2.30	32.26	255.62
All Clinics	203.61	33.53	5.38	110.78	1.24	37.09	391.65
All Facilities	129.81 (40.1%)	33.53 (10.4%)	4.74 (1.5%)	119.12 (36.8%)	1.77 (0.5%)	34.68 (10.7%)	323.63

### The costs of targeted active TB screening

Demand creation which precedes TaS4TB screening involves senior level health care workers from national and provincial levels (Table 5). The diagnosis cost for TaS4TB was twice that of standard of care, USD153 vs USD69. Major cost drivers were demand creation, chest X-ray and sample transportation to nearest diagnostic facility (USD89, USD33 and USD19 respectively.

## Discussion

This study demonstrated that TDABC can be used as a management tool to understand services’ organisation, to map and compare resource utilization and cost in a public health program, and to identify opportunities to improve care delivery and lower cost. Key success enablers were strong orientation and training at the onset of the programme, joint development of protocol and tools, and use of nurses familiar with the TB processes. Zimbabwe delivers the primary health care with well-trained HCWs at the lowest level of care to manage the most common diseases affecting the local population [9]. Nurses in Zimbabwe are trained to manage commonly occurring diseases, including TB. Additionally, the collaboration between local partners, GF and the HBS team built local capacity for implementation.

We observed minimal variations in the workflow for treating TB and related comorbid conditions, HIV and diabetes. Lessons drawn from this study, therefore, can be applied to the rest of centres that provide TB services. The urban polyclinics had limited capacity in diagnosing, yet the volume of patients on treatment was similar to hospitals [10].

Treatment cost at urban polyclinics was more expensive than at district and provincial hospitals. Some of this difference was due to the higher compensation paid to HCWs in polyclinics, which were supported by the Ministry of Local Government, compared to the HCWs in hospitals, supported by the MoHCC. Despite donor funded retention allowances for rural hospitals, those HCW salaries were still lower than in urban polyclinics. The skill-mix also varied between clinics and hospitals. Higher paid nurses provided TB/HIV counselling care in urban polyclinics while lower paid lay counsellors provided these services at hospitals. Hospital staff generally operated at the top of their license, with nurses delivering direct patient care and lower-paid staff performing less-skilled roles. Nurses at clinics, however, performed multiple functions because of staffing gaps caused by the inadequate funding for many clinics. Third, hospitals mainly diagnosed TB patients and then referred them to clinics for treatment and follow up. Clinic TB diagnostic costs were 1·2 times higher than at hospitals because of the high specimen transport costs. HIV testing cost was also higher in clinics than hospitals, likely due to task shifting. Hospitals used lay personnel for HIV testing services while nurses performed HIV testing at clinics.

Ongoing treatment at clinics involved many steps, including bi-weekly treatment follow-ups, recording and reporting, specimen transportation costs, laboratory monitoring of treatment response, and medicines pick up. Zimbabwe could reduce costs by shifting to monthly follow-up visits, a frequency already used for antiretroviral therapy (ART) for HIV patients, and with treatment outcomes comparable to other low- and middle-income countries (LMICs) [11].

The NTP could also conduct a study on the cost-saving benefits from eliminating repeat sputum microscopy tests at the 5th and 6th monthly visits. The study would assess quality of care, misclassification errors of cured into treatment completed and proportion of missed multidrug resistant TB cases post-treatment. The cost data from this study was used for the 2020–2024 national strategic plan, using the TB/HIV Impact Measure and Estimates (TIME) modelling tool.

Cost of confirmation at Tas4TB was 2^·^2 times more than standard of care due to sample transportation costs and demand creation. Since Tas4TB was a new concept, opportunities to improve its efficiency are possible. For example, reorganising the Tas4TB workflow and reassigning of responsibilities from national level to district health care workers, especially for demand creation, would lower total costs. Embedding a molecular diagnostic equipment in the van used for Tas4TB would also lower costs. Optimising screening by Tas4TB is critical because studies from Zimbabwe and other countries have shown that targeting high risk groups increases access and reduces TB incidence [12, 13].

An inherent limitation of the study, due to it being the first to use TDABC to measure the cost of TB treatment, was the inability to compare and learn from treatment processes and costs in other low-to-medium income countries. In addition, a more longitudinal study to follow a cohort of patients through diagnosis to treatment completion would be able to provide more strong evidence.

Our study results provided data to inform possible policy changes and cost reductions. The cost of laboratory (diagnostic) services could be reduced by redeploying GeneXpert machines to reduce transport costs and improve access of TB diagnostic services to patients. This would require a thorough mapping exercise to assess the current reach of GeneXpert machines. Such a policy change would increase access for patients, reduce the cost of transport, and increase the capabilities (resources) and decrease processing time at each facility. The treatment sites could also batch TB diagnosis samples with those of disease programmes to optimize shared costs. Patients’ follow-up visits during treatment phase could be reduced from 12 to 6 by customizing drug-refill frequencies to patient risk characteristics. The reduction would lower provider costs and increase patient compliance, by removing repetitive process steps that do not lead to better treatment outcomes.

## Conclusion

The study has shown the feasibility of applying and embedding TDABC as a management tool for analysing and costing care processes in limited-resource public health programs. The study identified processes that would benefit from re-engineering, shifts in the skill mix of personnel used to perform tasks, and unpacked dimensions in domestic funding that were not visible. Performing drug refills for co-infected TB/HIV patients on the same schedule would reduce costs to both providers and patients. The dimensions of domestic funding that are not visible in the NSPs are contributions through the Zimbabwe Ministry of Local Government.

## Data Availability

The datasets used and/or analysed during the current study are available on a reasonable request to the first author, Dr. Joconiah Chirenda, joconiahc@gmail.com.

## Abbreviations

ART: Antiretroviral therapy
CCR: Capacity Cost Rate
Digital CXR: Digital Chest X-Ray
FBC Machine: Full Blood Machine
HCW: Health Care Workers
HIV: human immunodeficiency viruses
MDR-TB: Multi-drug resistant tuberculosis
MoHCC: Ministry of Health and Child Care
NSP: National Strategic Plan
Tas4TB: Targeted screening for TB
TDABC: Time Driven Activity Based Costing
TSH Machine: T*hyroid Stimulating Hormone*
TB: Tuberculosis
TB/HIV: Tuberculosis/Human immunodeficiency virus
